# Thickness-dependent photoelectrochemical properties of a semitransparent Co_3_O_4_ photocathode

**DOI:** 10.3762/bjnano.9.228

**Published:** 2018-09-12

**Authors:** Malkeshkumar Patel, Joondong Kim

**Affiliations:** 1 Department of Electrical Engineering, Incheon National University, 119 Academy Rd. Yeonsu, Incheon, 22012, Republic of Korea; 2 Photoelectric and Energy Device Application Lab (PEDAL), Multidisciplinary Core Institute for Future Energies (MCIFE), Incheon National University, 119 Academy Rd. Yeonsu, Incheon, 22012, Republic of Korea

**Keywords:** cobalt(II,III) oxide (Co_3_O_4_), photocathode, photoelectrochemical cells, semitransparent, thickness-dependent properties

## Abstract

Co_3_O_4_ has been widely studied as a catalyst when coupled with a photoactive material during hydrogen production using water splitting. Here, we demonstrate a photoactive spinel Co_3_O_4_ electrode grown by the Kirkendall diffusion thermal oxidation of Co nanoparticles. The thickness-dependent structural, physical, optical, and electrical properties of Co_3_O_4_ samples are comprehensively studied. Our analysis shows that two bandgaps of 1.5 eV and 2.1 eV coexist with p-type conductivity in porous and semitransparent Co_3_O_4_ samples, which exhibit light-induced photocurrent in photoelectrochemical cells (PEC) containing the alkaline electrolyte. The thickness-dependent properties of Co_3_O_4_ related to its use as a working electrode in PEC cells are extensively studied and show potential for the application in water oxidation and reduction processes. To demonstrate the stability, an alkaline cell was composed for the water splitting system by using two Co_3_O_4_ photoelectrodes. The oxygen gas generation rate was obtained to be 7.17 mL·h^−1^ cm^−1^. Meanwhile, hydrogen gas generation rate was almost twice of 14.35 mL·h^−1^·cm^−1^ indicating the stoichiometric ratio of 1:2. We propose that a semitransparent Co_3_O_4_ photoactive electrode is a prospective candidate for use in PEC cells via heterojunctions for hydrogen generation.

## Introduction

Hydrogen production using water splitting in photoelectrochemical (PEC) cells may help to overcome challenges in the conversion and storage of solar energy. Most of the metal oxides are earth-abundant, non-toxic, stable and easy to synthesise, and hence attractive regarding low-cost and reliable PEC cells [1–8]. For a widespread application of PEC cells, the photoelectrodes need to fulfill the criteria of (i) a low band gap (1.7–2.2 eV), (ii) low resistivity, (iii) low cost, (iv) corrosion stability and (v) a correct alignment of band edges with respect to the water redox potential [3,9–10]. The spinel Co_3_O_4_ is interesting because of its dual bandgap (1.5 and 2.2 eV), high absorption coefficient, intrinsic p-type doping and chemical stability. It has found application as a light-absorbing entity in all-metal-oxide photovoltaic cells [11–17]. Dual-bandgap Co_3_O_4_ films provide distinct band states in the energy–momentum diagram, which is advantageous to reduce the thermalisation-related losses in the sunlight-driven hydrogen generation. Dual bandgaps in Co_3_O_4_ originate from the crystal-field split Co 3d states at the octahedral (Co^3+^) and tetrahedral (Co^2+^) cobalt sites, where Co vacancies are the dominant sources of the p-type conductivity of Co_3_O_4_ under oxygen-rich conditions [9,13]. Despite these interesting properties of Co_3_O_4_ its application in photocathodes has been rarely studied [18–23]. Existing studies have measured a photocurrent of 33.6 μA·cm^−2^ in 0.5 M Na_2_S on a mesoporous Co_3_O_4_ nanosheet grown through in situ transformation from hexagonal Co(OH)_2_ to spinel Co_3_O_4_ [18]. Hong et al. demonstrated a photocurrent of 0.4 mA·cm^−2^ from Co_3_O_4_ nanowire photocathodes, which could be enhanced to 4.5 mA·cm^−2^ with Ag nanowires [24]. Interestingly, a high photocurrent density of 29 mA·cm^−2^ can be achieved from Co_3_O_4_ under one-sun illumination (AM1.5G) suggesting a high (solar-to-hydrogen) efficiency of 35.8% [3].

Studies using Co_3_O_4_ as a catalyst have explored the oxygen evolution reaction (OER) [25–26] and the hydrogen evolution reaction (HER) [20,27] to prove its outstanding stability [28] for the use in water-splitting applications. It therefore may be applied as a protective heterojunction layer to overcome the typical overpotential in photoactive materials. Examples of materials used in such applications include Cu*_x_*O [19,29], CdS [30], TiO_2_ [31], Fe_2_O_3_ [32], and BiVO_4_ [33–34]. To absorb light with Co_3_O_4_, an adequately thick film is required. However, the low mobility of photogenerated charge carriers in Co_3_O_4_ can result in a low carrier lifetime, which is detrimental for efficient charge collection in photoactive applications [9,13,20]. In this context, relatively thin Co_3_O_4_ samples can overcome charge collection problems due to its semitransparency, which has been investigated in this study. To fabricate Co_3_O_4_ samples, Kirkendall diffusion is effective to induce the thermal oxidation of Co under atmospheric conditions, which provides an enhanced surface area due to porous features [19,32–38].

Our previous study on porous Co_3_O_4_ films grown by Kirkendall diffusion exhibited efficient photoelectrocatalystical seawater splitting due to its favourable HER properties [20]. We also developed compact Co_3_O_4_ films by a reactive sputtering method, in which sputtered Co particles were converted into a compact Co_3_O_4_ film by controlling the flowing O_2_ gas, to offer a self-powered ultraviolet photodetector [17] and semitransparent photovoltaics [39]. It is noteworthy to mention that Co_3_O_4_ films grown by Kirkendall diffusion have the advantages of a porous structure, a higher growth rate, and easy fabrication.

Here, we report thickness-controlled Co_3_O_4_ photoactive electrodes in PEC cells that include the water oxidation and the reduction potentials. We thermally oxidize Co nanoparticles in air that form a porous semitransparent Co_3_O_4_ layer through Kirkendall diffusion. The structural, physical, optical, electrical and photoelectrochemical properties of Co_3_O_4_ samples are presented as functions of the thickness. The alkaline cell was composed for water splitting by using two Co_3_O_4_ photoelectrodes. We propose a promising route for photoactive, semitransparent Co_3_O_4_ embedded in PEC cells for the light-driven hydrogen generation through water splitting.

## Results and Discussion

**Figure 1:**
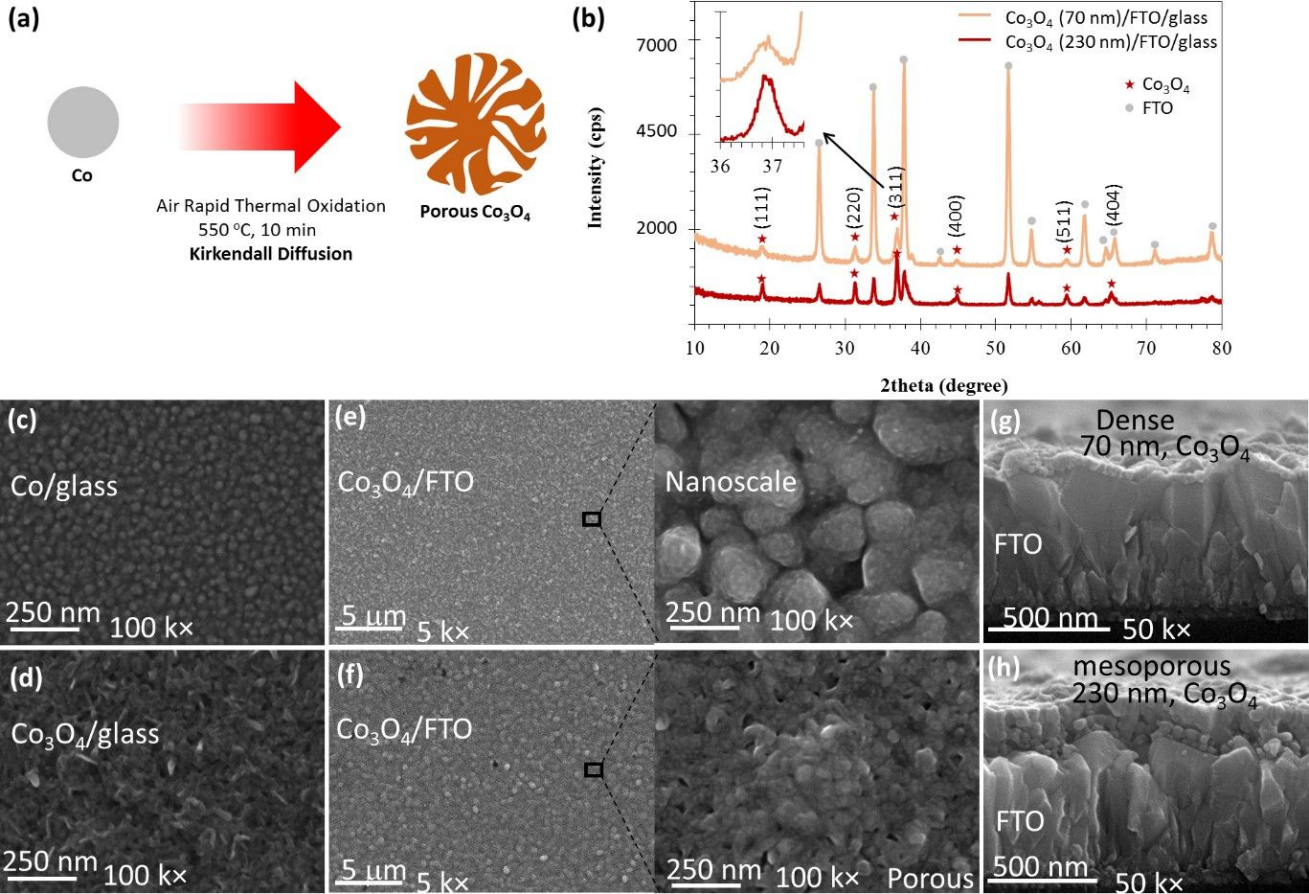
(a) Kirkendall diffusion-induced growth of porous Co_3_O_4_. (b) X-ray diffraction pattern of Co_3_O_4_ films prepared on the FTO/substrate. Inset shows a strong XRD peak that corresponds to the (311) planes of cubic Co_3_O_4_ at 36.81°. FESEM images showing (c) the topography of Co film deposited at room temperature on the glass substrate, and (d) the porous topography of Co_3_O_4_ on the glass substrate after rapid thermal processing-induced oxidation in air at 550 °C for 10 min. (e) Morphology of a 70 nm thick Co_3_O_4_ film on FTO/glass, (f) morphology of a 230 nm thick Co_3_O_4_ film on FTO/glass, cross-sectional images of (g) a thin Co_3_O_4_ (70 nm) film on FTO/glass substrate showing the densely packed film covering the FTO surface, and of (h) a thicker Co_3_O_4_ layer (230 nm) showing the mesoporous aspect attributed to the Kirkendall diffusion-driven thermal oxidation of the Co particles.

The oxidation of Co nanoparticles formed a porous Co_3_O_4_ structure due to the nanoscale Kirkendall effect as shown in Figure 1a, which arises from the difference in diffusion rates between the anions and cations [35,40]. We applied rapid thermal oxidation to sputtered Co nanoparticles in air at 500 °C for 10 min to convert them into Co_3_O_4_ [20]. Co films of varying thickness were deposited using large-area (4 inch diameter) sputtering on glass and FTO/glass substrates. Identical rapid thermal processing (RTP) oxidation was applied to these Co films to allow the formation of Co_3_O_4_ films of varying thickness and porosity.

Figure 1b shows the XRD pattern of two prepared Co_3_O_4_ samples, 70 nm and 230 nm thick, grown on the FTO/glass substrate. XRD confirmed the formation of a crystalline Co_3_O_4_ phase due to the air-induced diffusion-driven oxidation of Co. XRD peaks corresponding to Co_3_O_4_ and F:SnO_2_ (substrate) were identified and marked. A stronger XRD peak at 2θ = 36.81° corresponds to the (311) planes of cubic Co_3_O_4_ with a *d*-spacing of 2.411 Å, in agreement with the crystallographic open database file COD-9005888. According to this XRD pattern, the Co_3_O_4_ material has a lattice parameter of *a* = 8.09 Å (cubic, *a* = *b* = *c*). The XRD peaks at 18.90°, 31.20°, 44.73°, 59.30°, and 65.12° correspond to the (111), (220), (400), (511), and (404) crystal planes, respectively [20,41].

When compared to the F:SnO_2_ substrate material, the XRD peaks corresponding to the 230 nm thick Co_3_O_4_ sample are more intense. The absence of XRD peaks of pure Co indicates that the applied RTP fully oxidized the Co film into a Co_3_O_4_ film with controlled thickness and porosity, which is further validated below.

Figure 1c,d shows the surface morphology of both the deposited Co and the RTP-grown Co_3_O_4_ film on the glass substrate, respectively. FESEM results confirm that the deposited film contains spherical Co particles, and conversion into porous Co_3_O_4_ is attributed to Kirkendall-diffusion-induced thermal oxidation.

The planar morphology is shown in Figure 1e and Figure 1f, and corresponds to 70 nm and 230 nm thick Co_3_O_4_ films grown on the FTO substrate, respectively. The as-grown Co_3_O_4_ films are uniform and interconnected. A cross-sectional FESEM image of the 70 nm thick Co_3_O_4_ film, shown in Figure 1g, reveals compact and dense features, while the 230 nm thick Co_3_O_4_ film, seen in Figure 1h, reveals porous features uniformly distributed across the FTO surface. This subtle morphology change in the crystalline Co_3_O_4_ can be applied to grade its porosity by simply varying the Co thicknesses prior to thermal oxidation. Therefore, we prepared Co_3_O_4_ samples with varying thicknesses from 70 to 230 nm, which were extensively studied with regard to their optical, electrical, interfacial, and photoelectrochemical cell properties.

**Figure 2:**
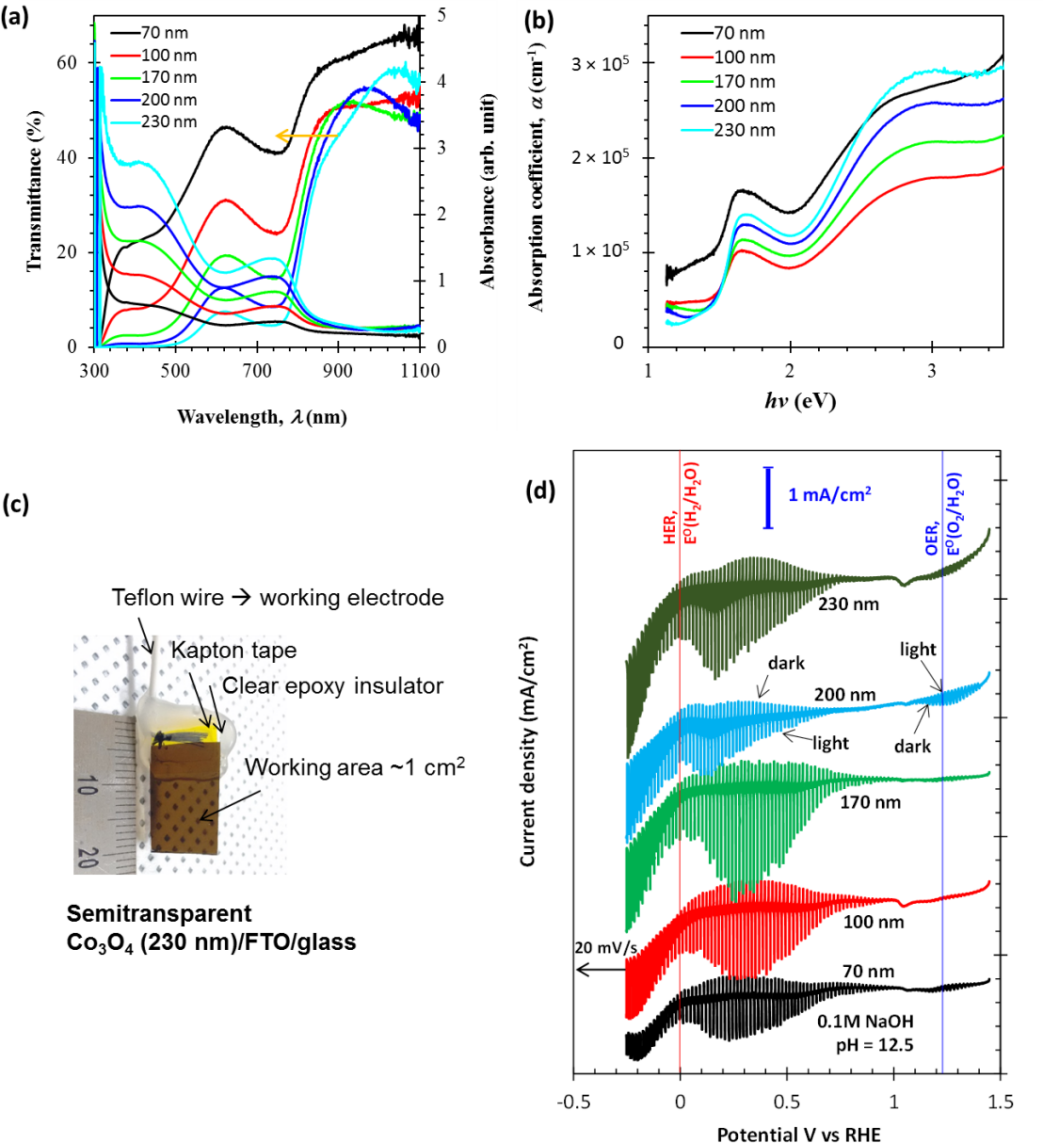
(a) Optical characteristics including the transmittance and absorbance spectra of Co_3_O_4_ films. (b) Absorption coefficient (α) as a function of the photon energy (*hv*). (c) Photograph of a Co_3_O_4_ electrode for photoelectrochemical cell studies. (d) Linear sweep voltammogram (from 1.45 to −0.28 V vs RHE) of Co_3_O_4_ electrodes under pulsed light (100 mW·cm^−2^).

Figure 2a shows the thickness-dependent transmittance (*T*) and absorbance (*A*) spectra of the Co_3_O_4_ samples. Interestingly, all the Co_3_O_4_ samples exhibited fair absorbance with semitransparent optical properties. Absorbance dominates the shorter wavelengths (λ = 300–500 nm) with transmittance dominating at longer wavelengths from 600 nm to the infrared (IR). Two distinct transitions in both the *T* and *A* spectra of all Co_3_O_4_ samples are attributed to two bandgaps coexisting in the Co_3_O_4_ material. A 70 nm thick Co_3_O_4_ sample exhibited a higher *T* in the IR region than the other Co_3_O_4_ samples, which is attributed to its dense and compact film, which causes lower absorption of free carriers than the porous surface.

The absorption coefficient (α), which determines the absorption length of the Co_3_O_4_ samples was estimated using the relation


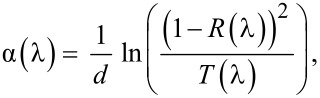


where *d* and *R* are the thickness of the Co_3_O_4_ layer and the reflectance, respectively. Figure 2b shows α estimated as a function of photon energy (*hν*). The influence of the Co_3_O_4_ morphology on α is interesting. Except for the 70 nm film, α increases with thickness, suggesting an increase in porosity as well. A higher porosity led to higher α values, which are prominent in the 440–350 nm region. This feature is useful for a porous Co_3_O_4_ material as a semitransparent electrode in water-splitting PEC cells. Moreover, two distinct transitions (positive slope) around 1.5 and 2.4 eV in Figure 2b have different α values, providing optical selectivity that can be controlled by film thickness.

Having identified this useful semitransparency of Co_3_O_4_, we prepared working electrodes to study the thickness-dependent performance of the photoelectrochemical cell. Figure 2c shows a semitransparent Co_3_O_4_ working electrode with an active area of 1 cm^2^. Clear epoxy resin was applied to the rest of the surface to prevent an electrical connection to the working terminal of the potentiostat/galvanostat. Co_3_O_4_ has evolved as a chemically resistive and stable material for electrolysis reactions [20,25–27], and therefore the potential of the Co_3_O_4_ working electrode was swept from 1.5 to −0.3 V vs RHE in a 0.1 M NaOH electrolyte (pH 12.5). The thickness-dependent linear sweep voltammogram (LSV) of the PEC cell under chopped light illumination is shown in Figure 2d. These results provide an overview of the photoinduced OER at 1.23 V vs RHE, the HER at 0 V vs RHE, and the operation of the photocathode. The value of the onset potential (*V*_on_), which is the condition attributed to a minimum charge transfer of the cell, was found to be just below that of the OER potential. All of the Co_3_O_4_ samples exhibited a photoresponse, suggesting photoabsorption and the utilization of photogenerated charges in the PEC cells. A strong thickness dependence on the photoresponse was found and showed that thicknesses of 100–170 nm are adequate to gain significant photocurrents. More interestingly, the 170 nm Co_3_O_4_ sample exhibited maximum photocurrent values in the applied potential region of 0.2–0.4 V vs RHE as described below in detail. It is fundamental to optimise the thickness of the Co_3_O_4_ film. In order to improve light absorption, a thicker film is better. However, the Co_3_O_4_ film has a short carrier diffusion length due to the slow electron extraction kinetics, resulting in a degraded conversion efficiency [14]. This is the reason for the current decrease for the relatively thick Co_3_O_4_ films from 170 to 230 nm.

**Figure 3:**
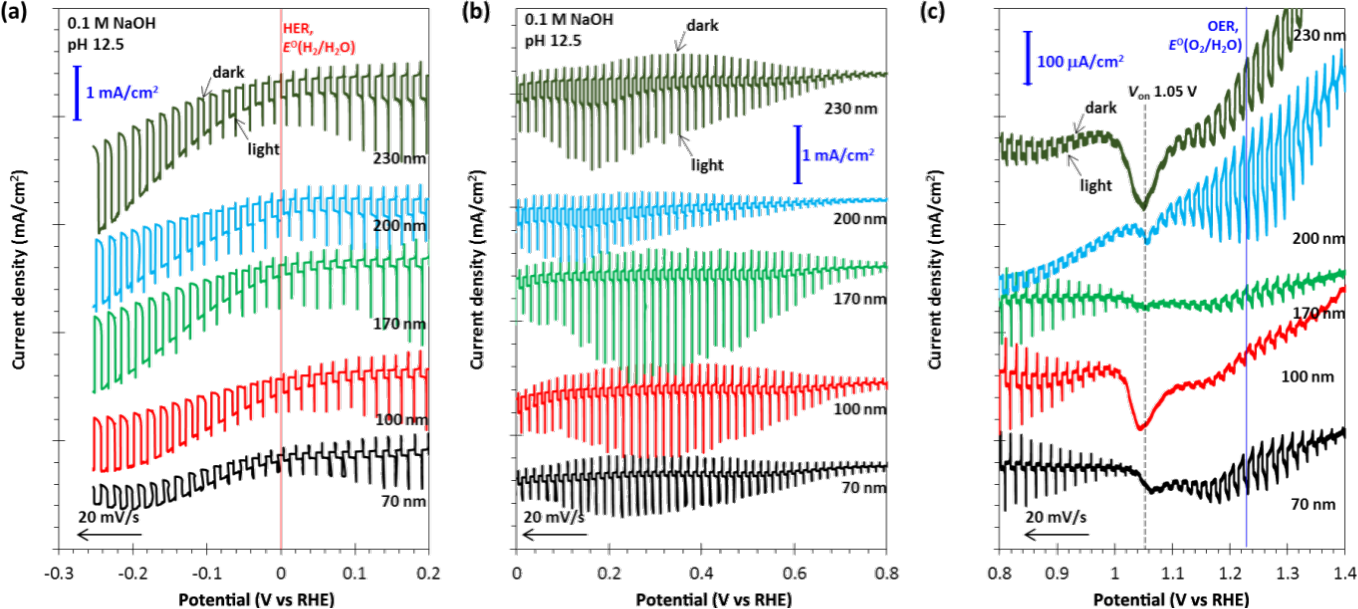
Thickness-dependent linear sweep voltammetry of a Co_3_O_4_ working electrode under pulsed light. (a) 0.2 to −0.3 V vs RHE (inversion to deep inversion condition as well as HER); (b) 0.8 to 0 V vs RHE (Co_3_O_4_ undergoing flat band to depletion condition); (c) 1.4 to 0.8 V vs RHE (covering the onset potential, which is close to the flat band potential as well as OER).

The thickness-dependent LSV for the potentials applied to the Co_3_O_4_ photoelectrode includes the water oxidation and reduction potentials as shown in Figure 3a–c. The photoresponse of the Co_3_O_4_ samples in the potential range from −0.25 to 0.2 V vs RHE corresponds to the hydrogen evaluation reaction as shown in Figure 3a. The Co_3_O_4_ samples with thicknesses from 100 to 230 nm showed identical photocurrent values of ca. 1 mA·cm^−2^. An increased dark-current level, indicating catalytic properties of the Co_3_O_4_ material, may have an advantage as the photoinduced current is of great interest in achieving photoinduced water reduction reactions in PEC cells.

When the PEC cell containing the Co_3_O_4_ working electrode went to the depletion region from a flat band condition, the photoresponse was prominent, as shown in Figure 3b. The low dark current (*J*_d_), which is consistent throughout the potential from 0.8 to 0.1 V vs RHE, confirms the chemical stability of the Co_3_O_4_ material. Meanwhile, these results also characterized the photoactive properties of the depletion region and its modulation in the Co_3_O_4_ electrode. The height of the spikes seems to be related to the bias voltage.

Figure 3c shows the LSV photoresponse near the onset potential of 1.05 V vs RHE, the *V*_on_ region, for all the samples confirming that the variation in thickness and porosity do not affect *V*_on_. However, thicker samples exhibited a higher photocurrent density in the anodic region, which can be interesting for studying the possibilities of light-induced water oxidation reactions. In this context, specifically, the Co_3_O_4_ film with a thickness of 200 nm showed a photocurrent density (*J*_photo_) up to 120 μA·cm^−2^ at 1.23 V vs RHE.

**Figure 4:**
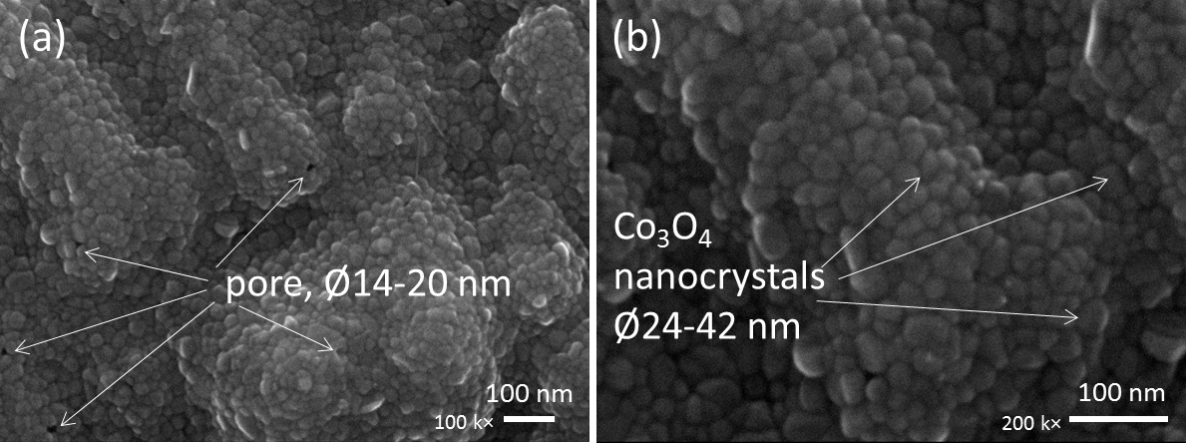
Surface morphology of the 170 nm thick Co_3_O_4_ film on FTO/glass showing (a) the pores with diameters of 14–20 nm and (b) Co_3_O_4_ nanocrystals with diameters of 24–42 nm.

For precise observation, we provided the morphologies of the 170 nm thick Co_3_O_4_ film in Figure 4. The FESEM images clearly showed the pores (diameter 14–20 nm) and the nanocrystals (diameter 24–42 nm). Through the pores the surface area of Co_3_O_4_ film can be enhanced. Meanwhile, the nanocrytals work as the efficient routes for charge collection.

Next, the current–time characteristics of the 170 nm thick semitransparent Co_3_O_4_ photocathode were studied in transient light as shown in Figure S1 (Supporting Information File 1). Chronoamperometry studies at 0 V vs RHE for the Co_3_O_4_ photocathode show an initial *J*_photo_ = 0.7 mA·cm^−2^ that stabilized to 0.55 mA·cm^−2^ after 30 min of operation, demonstrating a stable PEC cell operation. This also indicates that porous Co_3_O_4_ can be a candidate for a semitransparent photocathode as a chemically stable and optically active material.

**Figure 5:**
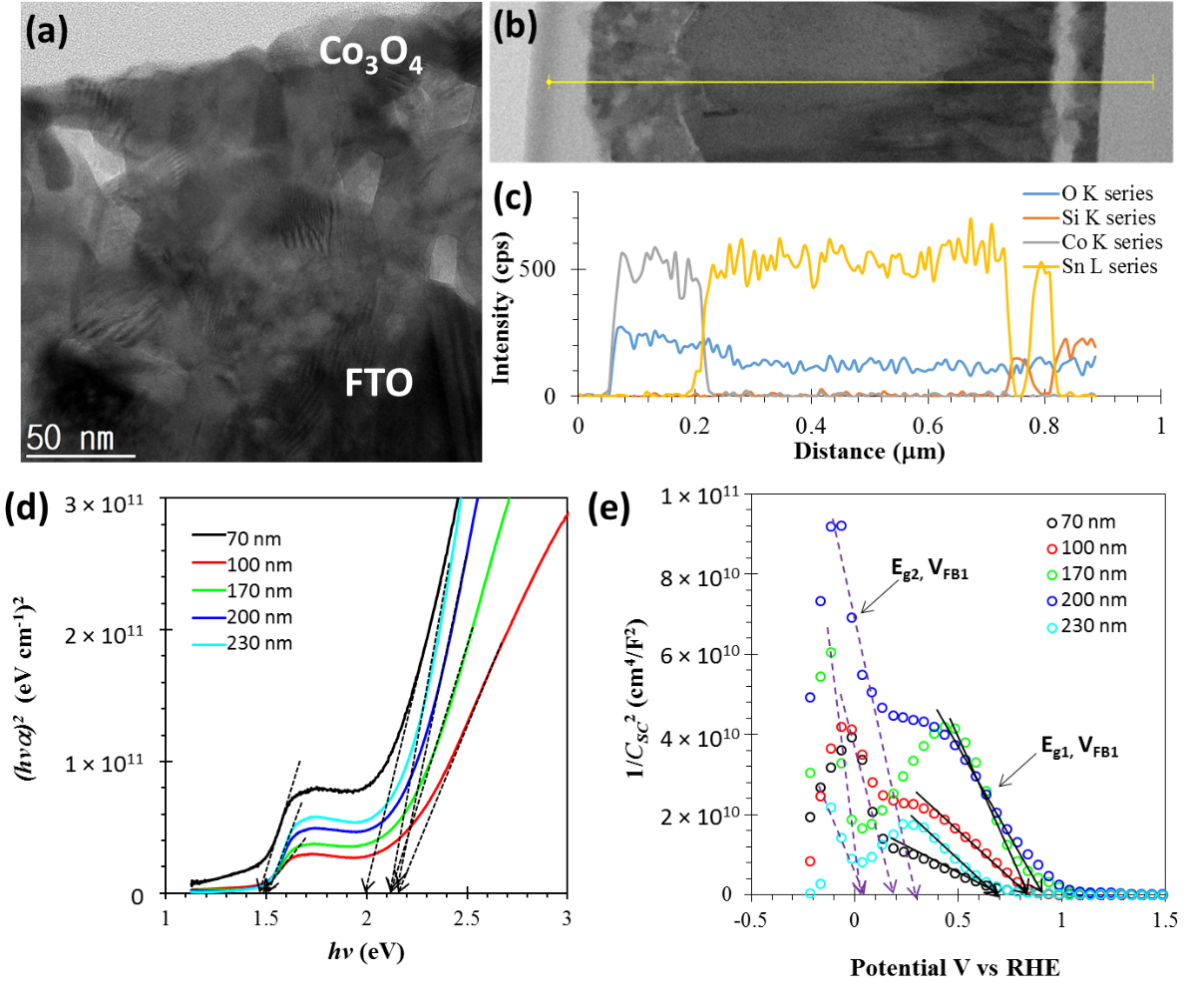
(a) Transmittance electron micrograph featuring nanocrystalline features of a Co_3_O_4_ electrode prepared on FTO/glass. (b) Cross-sectional image and (c) elemental line profile of Co_3_O_4_/FTO/glass electrode. (d) Tauc’s relation showing the values of two direct bandgaps in Co_3_O_4_ and their dependence on the thickness of the film. (e) Thickness-dependent Mott–Schottky characteristics of Co_3_O_4_/FTO electrodes.

In order to better understand the photoactivity and semitransparency of the Co_3_O_4_ photocathode, transmission electron microscopy was performed on the 170 nm thick Co_3_O_4_ sample as shown in Figure 5a–c. The cross-sectional TEM image of the Co_3_O_4_ photocathode seen in Figure 5a shows the flawless Co_3_O_4_/FTO interface, which is desirable for efficient transport of photogenerated charges. Moreover, the TEM image shows the nanocrystalline nature of the porous Co_3_O_4_ due to the Kirkendall diffusion that drove the thermal oxidation of Co nanoparticles on the FTO layer. The bright-field distribution observed from the TEM image further illuminates the porous features of the Co_3_O_4_ nanocrystals and the enhanced photocurrent in the PEC cell performance.

A complete cross-sectional image of the Co_3_O_4_/FTO/glass using the TEM is shown in Figure 5b. This confirms a void-free interface, which is typically difficult to obtain in samples grown using Kirkendall diffusion oxidation, as it generally leads to a core–shell structures. However, here it yielded a porous Co_3_O_4_ film that can be applied in water-splitting devices. An elemental line profile garnered from energy dispersive spectroscopy as shown in Figure 5c supports the claim of porosity in the grown Co_3_O_4_ film and the void-free interface between Co_3_O_4_/FTO.

Further, we estimated the thickness-dependent band-gap energies (*E*_g_) of the Co_3_O_4_ samples using Tauc’s relation as shown in Figure 5d. The coexistence of bandgaps two distinct band gaps with direct *E*_g_ values of around 1.5 and 2.1 eV is confirmed. Due to the porous and nanocrystalline nature of the Co_3_O_4_ samples, a blueshift in the *E*_g_ values is seen, compared to the dense 70 nm thick Co_3_O_4_ sample. Table 1 shows the summarized thickness-dependent optical and electrical properties of the Co_3_O_4_ samples.

**Table 1:** Summarized properties of the Co_3_O_4_/FTO samples. Here *t*, *T*, λ, *E*_g_, *V*_FB_ and *N*_A_ are the thickness of the Co_3_O_4_ layer, transmittance, photon wavelength, band gap, flat-band potential, and acceptor carrier concentration, respectively.

*t* (nm)	*T* (%)		*E*_g1_ (eV)	*E*_g2_ (eV)	*V*_FB1_ (V vs RHE)	*V*_FB2_ (V vs RHE)	*N*_A1_ (cm^−3^)	*N*_A2_ (cm^−3^)
	λ = 820 nm	λ = 560 nm						

70	53	39	1.45	2	0.7	0.18	4.9 × 10^20^	6.1 × 10^19^
100	41	24	1.47	2.16	0.84	0.32	2.5 × 10^20^	9.7 × 10^19^
170	35	15	1.49	2.14	0.82	0.06	8.8 × 10^19^	5.0 × 10^19^
200	29	8	1.5	2.13	0.93	0.3	1.4 × 10^20^	5.3 × 10^19^
230	25	4	1.51	2.12	0.7	0.08	2.5 × 10^20^	1.1 × 10^20^

Mott–Schottky (MS) characteristics allow us to describe the type of conductivity, free carrier concentration, and flat-band potential (*V*_FB_) of the samples. Figure 5e shows the thickness-dependent MS characteristics (1/*C*^2^ as a function of V vs RHE) of the Co_3_O_4_ samples, obtained at an applied frequency of 5 kHz and under dark conditions. The negative slope in the MS characteristics indicates a p-type material, and the two distinct slopes correspond to two *E*_g_ values. The intersect of the 1/*C*^2^ values on the potential axis indicates the flat-band potential, for which band edges are flat and PEC cells under this condition exhibit the minimum charge transfer. Additional details on the identification of band edges and *V*_FB_ in the Co_3_O_4_ samples can be found elsewhere [20]. Figure S2 and Figure S3 (Supporting Information File 1) provide analysis of the MS characteristics including the values of *N*_A1_, *N*_A2_, *V*_FB1_, and *V*_FB2_, which are also summarized in Table 1, where *N*_A_ is the acceptor carrier concentration. Thickness-dependent parameters including the *T*, *E*_g_, and *N*_A_ values of the Co_3_O_4_ samples suggest that the enhanced photocurrent performance of the PEC cell containing a 170 nm thick Co_3_O_4_ film are primarily due to its enhanced porosity and optical absorption. We also studied the thickness dependent optical and electrical properties of Co_3_O_4_ film grown by reactive sputtering [17]. In fact, we can see the systematic variation of Mott–Schottky characteristics, and so of the *V*_FB_ and *N*_A_ values of the samples grown by Kirkendall diffusion. This variation is attributed to the varying porosity that does not occur shown in the compact Co_3_O_4_ film [20].

**Figure 6:**
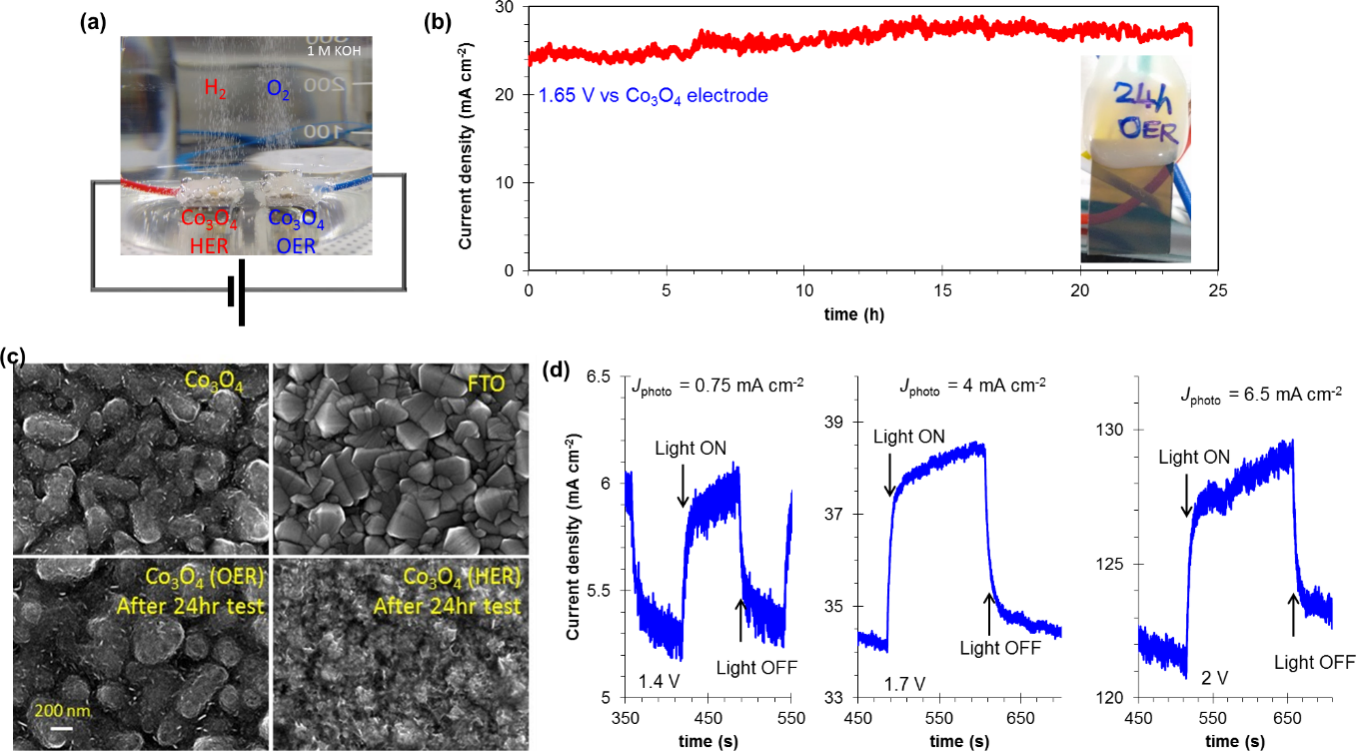
(a) PEC cell setup by using dual Co_3_O_4_ electrodes to show O_2_ gas generation in OER side and H_2_ gas generation in HER side. (b) Current stability test for 24 h at a bias of 1.65 V vs Co_3_O_4_ electrode (inset is the photograph of the Co_3_O_4_ electrode after 24 h test). (c) FESEM images after test. (d) Current–time plots (at bias values of 1.4, 1.7, and 2 V vs Co_3_O_4_ electrode) under pulsed light illumination.

In order to investigate the long-term stability, the PEC cell was tested for 24 h as shown in Figure 6a. The PEC cell has dual Co_3_O_4_ electrodes with a potential of 1.65 V vs a Co_3_O_4_ electrode in 400 mL of an alkaline bath (1 M KOH). The measured current value is presented in Figure 6b for a current density of 25 mA·cm^−2^ that is stable over a period of 24 h. In order to see the morphological changes after 24 h, the Co_3_O_4_ electrodes were observed by using FESEM as shown in Figure 6c. The OER side of the Co_3_O_4_ film seems to be similar to a pristine film. The HER side of the Co_3_O_4_ electrode is also in good shape. As a reference, the FTO image is also presented.

Further, varying bias values (1.4, 1.7 and 2 V) were applied to monitor the water-splitting reaction. Figure 6d shows bias-dependent current profiles. At 1.4 V a current density of 5.25 mA·cm^−2^ with a photocurrent density of 0.75 mA·cm^−2^ under illumination was observed. With an enhanced bias of 2 V, a significantly enhanced photocurrent density (6.5 mA·cm^−2^) was obtained. This result clearly shows the potential of the Co_3_O_4_ electrode to achieve high photocurrents at a realtively low potential value. The obtained photocurrent density value for overall water splitting from dual Co_3_O_4_ electrodes in alkaline bath is more efficient in terms of the required overpotential than the seawater splitting (Co_3_O_4_||Pt electrodes) in our previous report [20].

**Figure 7:**
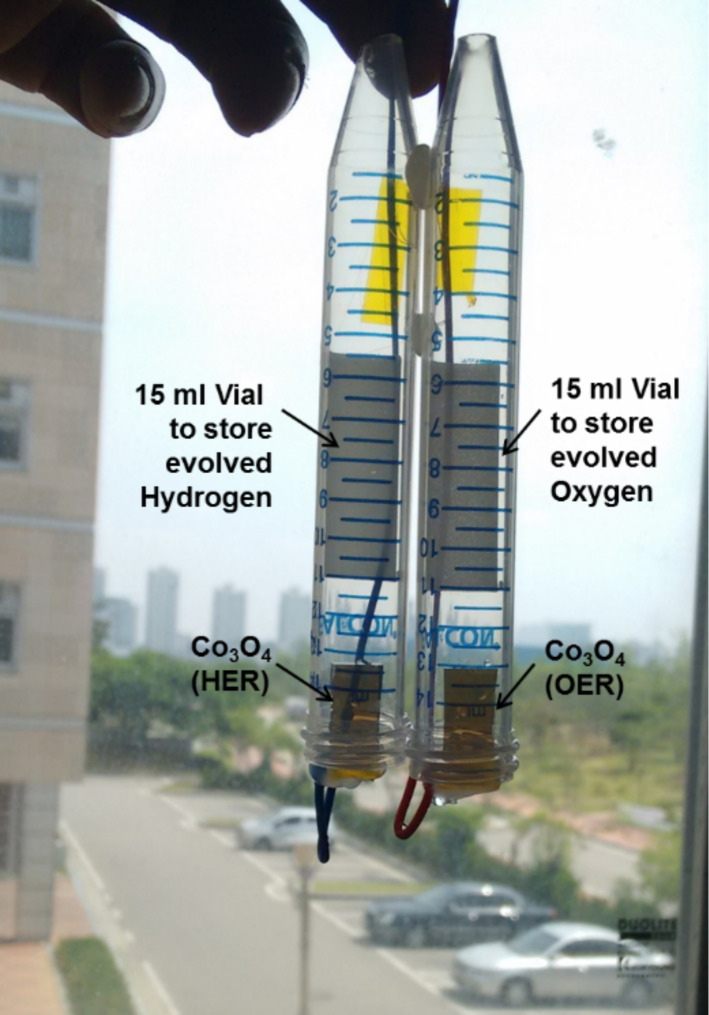
PEC cell setup with dual Co_3_O_4_ electrodes for volumetric measurements.

**Figure 8:**
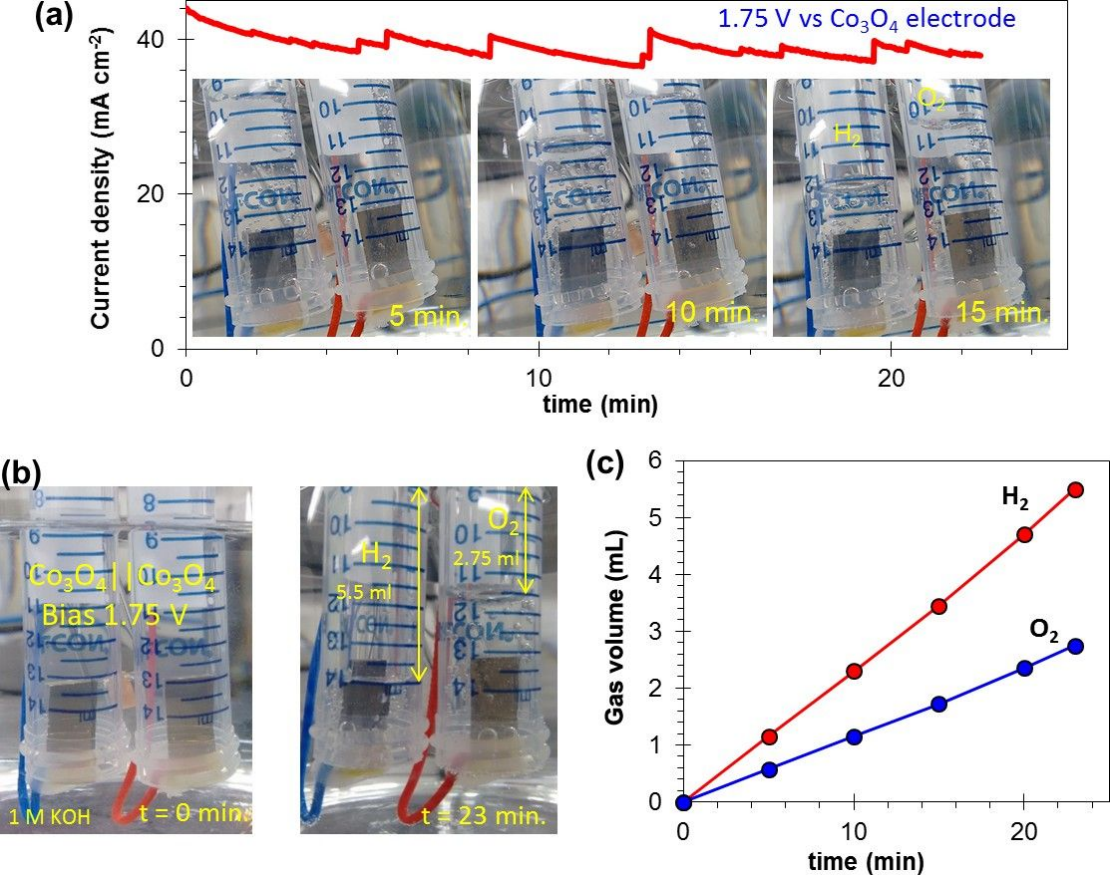
(a) Current density as a function of the time. The Co_3_O_4_ electrode was biased at 1.75 V in 1 M KOH electrolyte. Inset shows the photograph of gas evolution by time. (b) Photographs of the gas evolution at *t* = 0 and *t* = 23 min. (c) The hydrogen and oxygen gas evolution as a function of the time.

In order to verify the PEC performance, a PEC cell with dual Co_3_O_4_ electrodes was set up for volumetric measurements. The Co_3_O_4_ electrodes were loaded into two seperated vials (15 mL, Figure 7) and placed into the 1 M KOH electrolyte bath as shown in Figure 8a. A potential of 1.75 V was supplied to the Co_3_O_4_ electrode. The Co_3_O_4_ electrodes exhibited stoichiometric water splitting with an average current density of 39.5 mA·cm^−2^.

Evolution of the gases was clearly monitored in the two vials as presented in Figure 8b. After 23 min, 5.5 mL of hydrogen and 2.75 mL of were accumulated, corresponding to the ratio of 2:1 of water splitting. The hydrogen and oxygen gas evolution as a function of the time is presented in Figure 8c. The results show a hydrogen gas generation rate of 14.35 mL·h^−1^·cm^−1^ and an oxygen generation rate of 7.17 mL·h^−1^·cm^−1^ at a bias of 1.75 V vs Co_3_O_4_ electrode.

Our results demonstrate a stable photoinduced PEC cell performance with a semitransparent Co_3_O_4_ material made through an easy fabrication process. This could be of great interest for improving the water-splitting performance of emerging, earth-abundant light-absorber materials such as metal sulfides and metal oxides via heterojunction. The photocurrent can be further improved by three approaches: The first one is to improve the optoelectronic processes in the Co_3_O_4_ film [39], the second is to improve the composition of the heterojunction, i.e. Co_3_O_4_/Ga_2_O_3_ [42–43], and the third is the combination with a catalyst such as NiMo and transition-metal dichalcogenide 2D materials [43–44].

## Conclusion

We fabricated porous, semitransparent Co_3_O_4_ working electrodes of varying thickness using Kirkendall diffusion thermal oxidation in air. The thickness-dependent structural, physical, optical and electrical properties of the porous Co_3_O_4_ samples were studied. The application of a thickness-controlled Co_3_O_4_ film in a water-splitting PEC cell showed a light-induced photocurrent that included water oxidation and reduction processes. In particular, a photocurrent value of 1.5 mA·cm^−2^ corresponded to the reduction of the water when using a 170 nm thick Co_3_O_4_ sample. This sample provided enhanced photocurrent performance in the PEC cell, due to its enhanced porosity and absorbance. By using dual Co_3_O_4_ photoelectrodes, a hydrogen gas generation rate of 14.35 mL·h^−1^·cm^−1^ and an oxygen generation rate of 7.17 mL·h^−1^·cm^−1^ were obtained at a bias of 1.75 V vs Co_3_O_4_ electrode. The demonstration of a large-area, easy fabrication process to grow semitransparent Co_3_O_4_ samples would be pivotal for further application of light-driven water-splitting cells with heterojunctions.

## Experimental

**Sample fabrication:** The photocathode was composed of Co_3_O_4_/fluorine-doped tin oxide (FTO)/glass. The general synthesis of Kirkendall diffusion grown Co_3_O_4_ film was analogue to our previous study [20]. In brief, a commercial fluorine-doped tin oxide (FTO)-coated glass (735167, Sigma-Aldrich, sheet resistance of 7 Ω/sq) and a glass microscope slide were used as substrates. These were cleaned using a sequence of isopropyl alcohol, acetone and distilled water using ultrasonication. Then, various thicknesses of Co films were deposited using a dc magnetron sputtering system (dc power ca. 10 W·cm^−2^) was applied to a 4″ Co target (purity 99.99%). At a base pressure of 5 × 10^−5^ Torr sputtering gas (Ar) at a flow rate of 50 sccm was injected. To form the Co_3_O_4_ film, an atmospheric rapid thermal processing was applied at 550 °C for 10 min. The processing temperature was achieved in two stages. Ramp 1 increased the room temperature of 25 to 300 °C in 5 min. Ramp 2 then increased the temperature from 300 to 550 °C in 5 min. Natural cooling followed the RTP and at 100 °C samples were removed from the RTP chamber for characterization and electrochemical studies.

The Co_3_O_4_ working electrode was made of Teflon-coated wire that was applied to the FTO film with Kapton tape. Then, a clear insulating epoxy was applied to the Kapton tape and glass edges to provide a working area of 1 cm^2^.

**Materials characterization:** In order to examine the crystalline structure of Co_3_O_4_, an X-ray diffraction microscope (XRD, Rigaku, SmartLab) (Cu Kα radiation, λ = 1.540598 Å, in grazing mode with a glancing angle of 0.5°, step size of 0.05°, and a 2θ range of 10–80°) as well as a field-emission transmission electron microscope (FETEM, JEOL, JEM-2100F) were used. Cross-sectional TEM samples were prepared using a focused ion beam system (FIB, FEI, Quanta 3D FEG). The elemental compositions in the cross sections of the Co_3_O_4_ layers in the working electrode were determined as line profiles by an energy dispersive spectroscopy (EDS) attachment to the FETEM. Thickness and average surface roughness of the deposited films were characterized using a surface profiler (Vecco, Dektak XT-E). The planar and cross-sectional morphologies were analysed using a field-emission scanning electron microscope (FESEM, JEOL, JSM_7800F) with 5 kV of field voltage, using an SE2 secondary detector. Optical characterization was carried out using a UV–visible spectrophotometer (Shimadzu, UV-2600) by recording the transmittance, absorbance, and reflection of the Co_3_O_4_ films in the range of 300–1100 nm.

**Photoelectrochemical cell measurements:** Photoelectrochemical measurements were performed in a three-electrode cell with a potentiostat/galvanostat (PG-stat) (WonA Tech, ZIVE SP1). Co_3_O_4_/FTO/glass, Ag/AgCl (KCl, 3 M), and platinum gauze were connected to the working, reference, and counter electrodes of the PG-stat, respectively. All PEC cell measurements were carried out in 0.1 M NaOH aqueous electrolyte pH 12.5 at room temperature. A white light source (5800 K, Bridgelux, ES Star Array, BXRA-56C0700-A) with a light intensity of 100 mW·cm^−2^ was calibrated with a power meter (KUSAMMECO, KM-SPM-11). Scan rates of 20 mV·s^−1^ with a 0.1 mV step were set to record the linear sweep voltammetry (LSV), with the scan direction from positive to negative potentials in all cases. The measured potential vs Ag/AgCl were converted to the reversible hydrogen electrode (RHE) scale according to the Nernst relation, *E*_RHE_ = *E*_Ag/AgCl_ + 0.059 pH + *E*^0^_Ag/AgCl_, where *E*_RHE_ is the converted potential vs RHE, *E*_Ag/AgCl_ is the experimentally measured potential against the Ag/AgCl reference, and *E*^0^_Ag/AgCl_ = 0.210 V at 25 °C. A chronoamperometry (current–time characteristic) technique was applied at 0 V vs RHE to study the stability of the Co_3_O_4_ working electrode under pulsed light. In all of the photoinduced experiments, the Co_3_O_4_ surface was exposed to illumination. A Mott–Schottky (1/*C*_SC_^2^ as a function of V) analysis of the photoelectrodes was performed at an ac amplitude of 10 mV and in a frequency range from 5 kHz to 500 Hz. The dc potential was scanned from 1.4 to −0.4 V vs RHE with a sampling interval of 25 mV. All the PEC measurements were performed in an Ar purging environment at room temperature with 40 mL of electrolyte.

## Supporting Information

File 1 Additional experimental data.
