# Supplementary material for: Thickness-dependent photoelectrochemical properties of a semitransparent Co3O4 photocathode
Source: Beilstein J Nanotechnol. 2018 Sep 12;9:2432–42. doi: 10.3762/bjnano.9.228 (PMC6142771; doi:10.3762/bjnano.9.228)
Supplement: File 1 — Additional experimental data. [file Beilstein_J_Nanotechnol-09-2432-s001.pdf]

Supporting Information

for

Thickness-dependent photoelectrochemical properties of a semitransparent Co_3O_4 photocathode

Malkeshkumar Patel[1,2] and Joondong Kim*[1,2]

Address: [1]Department of Electrical Engineering, Incheon National University, 119 Academy Rd. Yeonsu, Incheon, 22012, Republic of Korea and [2]Photoelectric and Energy Device Application Lab (PEDAL), Multidisciplinary Core Institute for Future Energies (MCIFE), Incheon National University, 119 Academy Rd. Yeonsu, Incheon, 22012, Republic of Korea

Email: Joondong Kim - joonkim@incheon.ac.kr

*Corresponding author

Additional experimental data

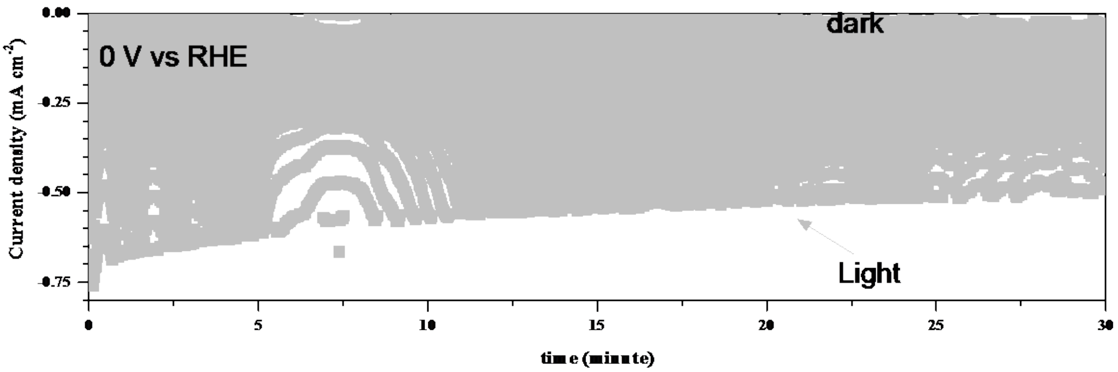

Figure S1: Chronoamperometry (current–time characteristic) of the 170 nm thick Co_3O_4 photoelectrode at 0 V vs RHE, under pulsed light.

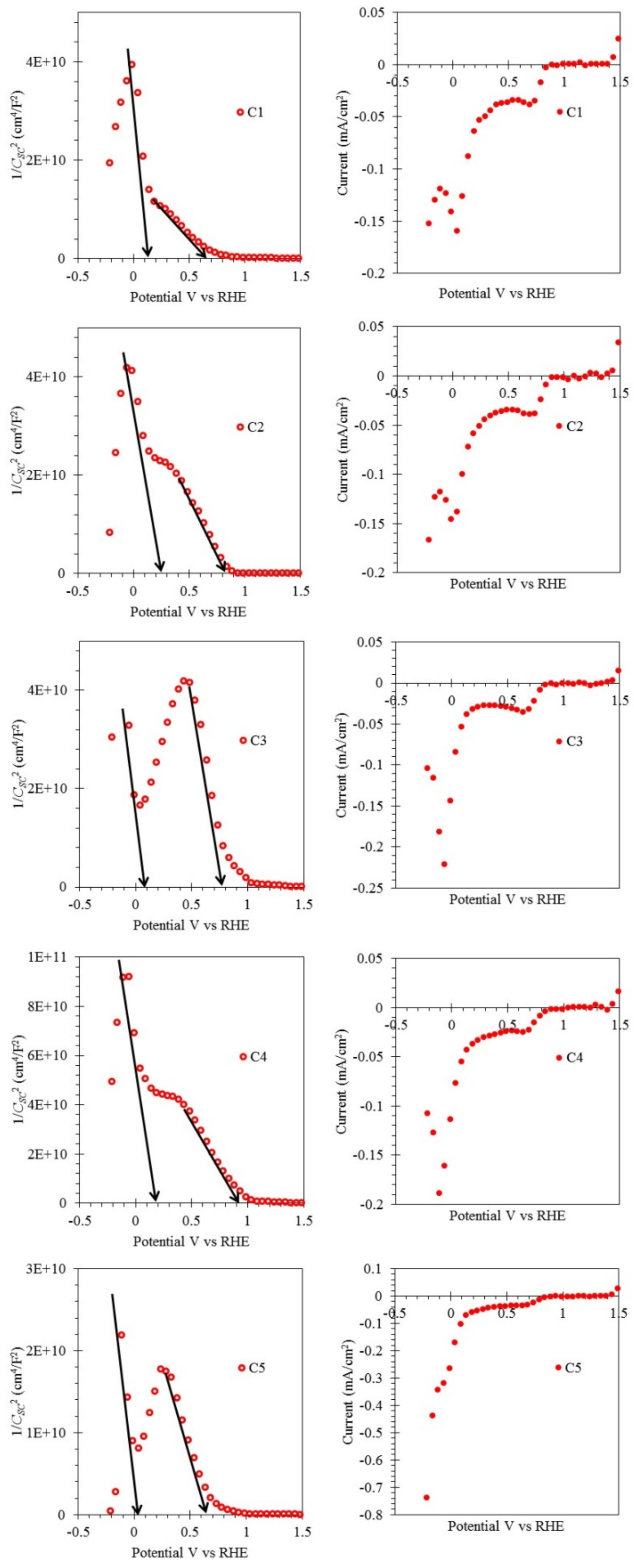

Figure S2: Thickness dependent Mott-Schottky characteristics of Co_3O_4 samples. Here C1, C2, C3, C4 and C5 refers to the Co_3O_4 film thickness of 70 nm, 100 nm, 170 nm, 200 nm, and 230 nm, respectively.

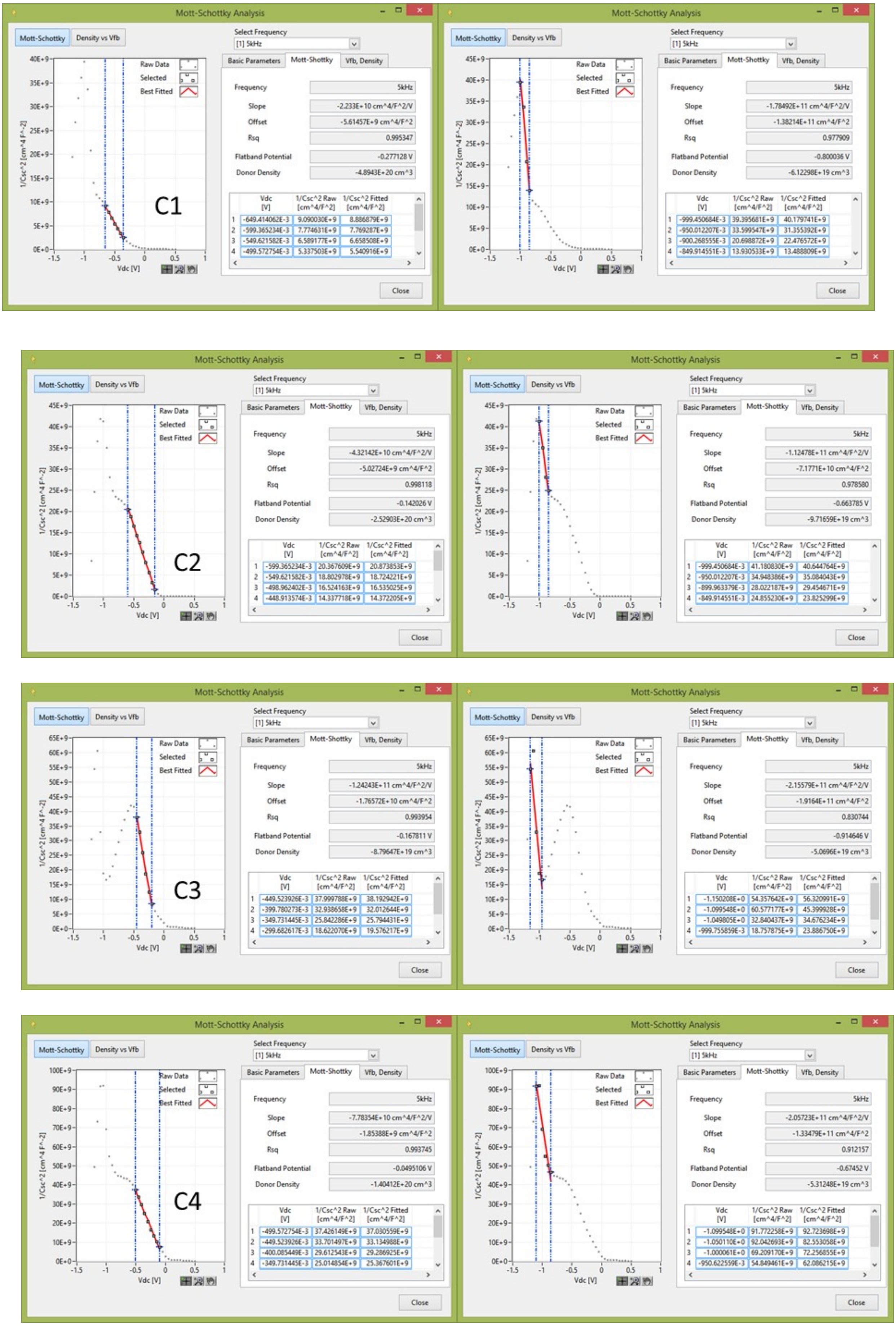

Mott-Schottky Analysis
C1
Slope -2.233E+10 cm^4/F^2/V
Offset -5.61457E+9 cm^4/F^2
Rsq 0.995347
Flatband Potential -0.277128 V
Donor Density -4.8943E+20 cm^3
Slope -1.78492E+11 cm^4/F^2/V
Offset -1.38214E+11 cm^4/F^2
Rsq 0.977909
Flatband Potential -0.800036 V
Donor Density -6.12298E+19 cm^3
C2
Slope -4.32142E+10 cm^4/F^2/V
Offset -5.02724E+9 cm^4/F^2
Rsq 0.998118
Flatband Potential -0.142026 V
Donor Density -2.52903E+20 cm^3
Slope -1.12478E+11 cm^4/F^2/V
Offset -7.1771E+10 cm^4/F^2
Rsq 0.978580
Flatband Potential -0.663785 V
Donor Density -9.71659E+19 cm^3
C3
Slope -1.24243E+11 cm^4/F^2/V
Offset -1.76572E+10 cm^4/F^2
Rsq 0.993954
Flatband Potential -0.167811 V
Donor Density -8.79647E+19 cm^3
Slope -2.15579E+11 cm^4/F^2/V
Offset -1.9164E+11 cm^4/F^2
Rsq 0.830744
Flatband Potential -0.914646 V
Donor Density -5.0696E+19 cm^3
C4
Slope -7.78354E+10 cm^4/F^2/V
Offset -1.85388E+9 cm^4/F^2
Rsq 0.993745
Flatband Potential -0.0495106 V
Donor Density -1.40412E+20 cm^3
Slope -2.05723E+11 cm^4/F^2/V
Offset -1.33479E+11 cm^4/F^2
Rsq 0.912157
Flatband Potential -0.67452 V
Donor Density -5.31248E+19 cm^3

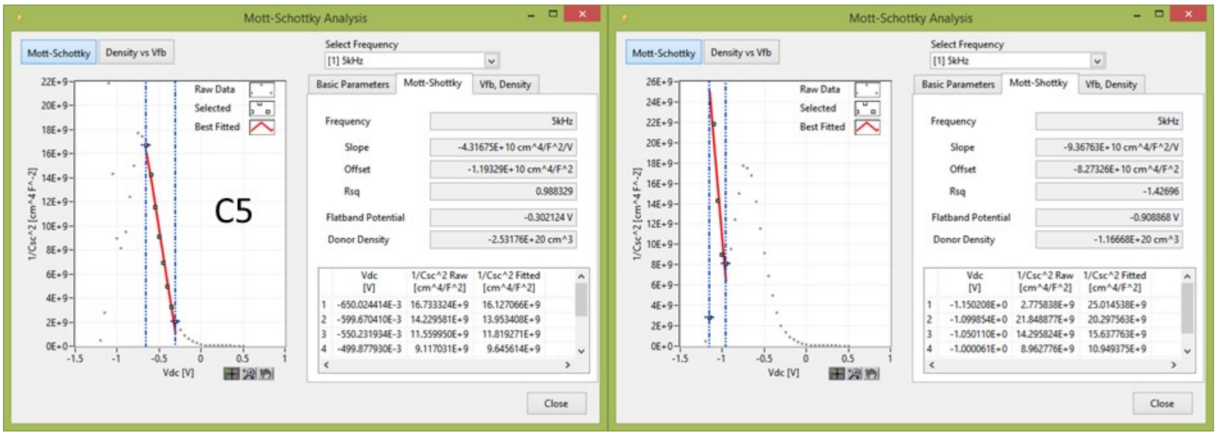

Figure S3: Analysis of Mott–Schottky characteristics of Co_3O_4 samples. Here C1, C2, C3, C4 and C5 refers to the Co_3O_4 film thickness of 70, 100, 170, 200 and 230 nm, respectively.
